# Berberine Mediated Positive Inotropic Effects on Rat Hearts *via* a Ca^2+^-Dependent Mechanism

**DOI:** 10.3389/fphar.2020.00821

**Published:** 2020-06-05

**Authors:** Junli Zhao, Yaqian Wang, Jie Gao, Yang Jing, Wenkuan Xin

**Affiliations:** College of Pharmaceutical Sciences, Southwest University, Chongqing, China

**Keywords:** berberine, heart, positive inotropic effect, Ca^2+^, Na^+^

## Abstract

Previous studies showed that berberine, an alkaloid from *Coptis Chinensis* Franch, might exert a positive inotropic effect on the heart. However, the underlying mechanisms were unclear. Here, we reported that berberine at 10–20 µM increased the left ventricular (LV) developed pressure and the maximal rate of the pressure rising, and it increased the maximal rate of the pressure descending at 20 µM in Langendorff-perfused isolated rat hearts. These effects diminished with the concentration of berberine increasing to 50 µM. In the concentration range of 50–300 µM, berberine increased the isometric tension of isolated left ventricular muscle (LVM) strips with or without electrical stimulations, and it (30–300 µM) also increased the intracellular Ca^2+^ level in the isolated LV myocytes. The removal of extracellular Ca^2+^ hindered the berberine-induced increases in the tension of LVM strips and the intracellular Ca^2+^ level of LV myocytes. These suggested that berberine might exert its positive inotropic effects *via* enhancing Ca^2+^ influx. The blockade of L-type Ca^2+^ channels (LTCCs) with nifedipine significantly attenuated 300 μM berberine-induced tension increase in LVM strips but not the increase in the intracellular Ca^2+^ level. Berberine (300 μM) further increased the LVM tension following the treatment with the LTCC opener FPL-64716 (10 μM), indicating an LTCC-independent effect of berberine. Lowering extracellular Na^+^ attenuated the berberine-induced increases in both the tension of LVM strips and the intracellular Ca^2+^ level of LV myocytes. In conclusion, berberine might exert a positive inotropic effect on the isolated rat heart by enhancing the Ca^2+^ influx in LV myocytes; these were extracellular Na^+^-dependent.

## Introduction

Berberine is an alkaloid present in numerous plants, including *Coptis Chinensis* Franch, and has been used for the treatment of gastrointestinal infections and diarrhea in Asian countries, particularly in China. The interest in its beneficial effects on significant risk factors of cardiovascular diseases has been growing over the last two decades (Lau et al., 2001; Zeng et al., 2003; Yuan et al., 2006; Affuso et al., 2010; Sola et al., 2014).

Berberine increased the developed force in the isolated left atrium of guinea pigs by enhancing both the force-velocity relationship and the duration of the active state (Shaffer, 1985). The contractile capability of the left ventricle is critical for heart function. As the left ventricle contracts, the pressure rises to a level that is high enough to pump blood into systemic circulation through the aorta (Bombardini, 2005). However, there is no study about the berberine effect on the LV contractility, which is vital for the pumping capability of the heart, and the underlying mechanisms by which berberine affects the left ventricle remains to be determined.

In LV myocytes, the Ca^2+^ entry through LTCCs triggers Ca^2+^ release from the sarcoplasmic reticulum into the cytoplasm. These consequently increase the intracellular Ca^2+^ level and cause LV myocytes shortening, which provides the force for the ejection of blood (Bers, 2002; Eisner et al., 2017; Gilbert et al., 2020). In addition to the Ca^2+^ influx through LTCCs, Ca^2+^ also enters the cell through an LTCC-independent pathway (Kiang and Smallridge, 1994; Tang et al., 1998). The Na^+^/Ca^2+^ exchanger (NCX) is the dominant transporter mediating Ca^2+^ efflux (Blaustein and Lederer, 1999; Philipson and Nicoll, 2000; Bers, 2002). The Na^+^ inﬂux during the early phase of an action potential induces Ca^2+^ influx *via* the reverse mode of NCX, which consequently induces the Ca^2+^ release from the sarcoplasmic reticulum and the elevation of intracellular Ca^2+^ level (Larbig et al., 2010; Yan et al., 2015).

In this study, we utilized Langendorff-perfused heart recordings, isometric tension recordings, and confocal microscopy techniques to investigate the effects of berberine on the LV contractility and the underlying mechanisms. Our study revealed that berberine elevated the intracellular Ca^2+^ level of LV myocytes *via* increasing Ca^2+^ influx. It consequently increased the LV contractility. These were extracellular Na^+^-dependent.

## Materials and Methods

### Ethics Statement

We carried out the animal procedures following The Guide for the Care and Use of Laboratory Animals of the National Institutes of Health, and the Institutional Animal Ethics Committee of Southwest University approved all the procedures.

### Animal Housing, Euthanasia, and Isolated Heart Harvesting

Adult male Sprague-Dawley rats (200–300 g) were purchased from Da Ping Hospital (Chongqing, China, SCXK 2017-0005) and were housed at Southwest University (Chongqing, China, SYXK 2014-0002). All rats were exposed to 12/12 h light/dark cycles at room temperature (24 ± 2°C) and were free to food and water. Rats were euthanized by CO_2_ inhalation or sodium pentobarbital injection (60 mg/kg body weight, ip), and the hearts were then quickly removed from the thoracic cavity by thoracotomy.

### Langendorff-Perfused Isolated Heart Recordings

Langendorff-perfused isolated heart recordings were carried out as previously described (Carre et al., 2014; Tilley et al., 2014). Briefly, rats were anesthetized with sodium pentobarbital (60 mg/kg body weight, ip), and the heart was rapidly excised by thoracotomy and then cannulated through the aorta. The isolated heart was mounted on a Langendorff apparatus (DMT MyoHEART 900MH System, Aarhus, Denmark) under a constant perfusion pressure (70 mmHg) and perfused with Krebs-Hensseleit (K-H) buffer saturated with a 95% O_2_/5% CO_2_ mixture, yielding a final pH of 7.4 at 37°C. For assessment of the performance of the left ventricle, a balloon connected to a pressure transducer was inserted into the left ventricle. It was used to set the left ventricular end-diastolic pressure (LVEDP) at 5–10 mmHg. After a 30-min stabilization period, berberine was added to the perfusion buffer with accumulative concentrations. Left ventricular systolic pressure (LVSP), LVEDP, LVDP (defined as *LVDP = LVSP—LVEDP*), the maximal rates of LVDP rising and descending during isovolumetric contraction and relaxation (+*dp/dt_max_* and |−*dp/dt*|*_max_*) were analyzed with LabChart 8.1.5 (ADInstruments, Australia).

### Isometric Tension Recordings

Isometric tension recordings in LVM strips were performed as previously described (Uhl et al., 2015). Briefly, the isolated hearts were transferred to the oxygenated K-H buffer containing 30 mM 2,3-butanedione monoxime (pH 7.4) at 4°C. LVM strips of 1–2 mm width, 1–1.5 mm thickness, and 6–8 mm length were prepared and mounted between metal clips attached to an anchoring hook and a force transducer in temperature-controlled (37°C) chambers filled with 10 ml K-H buffer and continuously bubbled with a 95% O_2_/5% CO_2_ mixture (DMT 750BTOS Tissue Organ bath system, Aarhus, Denmark). The strips were electrically driven with rectangular pulses (2 ms, 0.1 V, 1 Hz) using a CS4 stimulator (DMT instrument, Denmark). Pre-load was applied until the tension reached 5 mN, and then LVM strips were washed with fresh K-H buffer every 15 min during an equilibration period of 1 h. The contraction of LVM strips was induced by epinephrine (10 μM) or EFS (5 V, 1 Hz). Each berberine-treated strip had its vehicle control strip from the same ventricle.

### Left Ventricular Myocyte Isolation

The LV myocytes of a rat were isolated as described previously (Carre et al., 2014; Roth et al., 2014). Briefly, the isolated hearts were rapidly excised, cannulated through the aorta and retrograde perfused (5 ml/min, 37°C) with oxygenated Ca^2+^-free Tyrode's buffer using a Langendorff system (LGF-2C, Chengdu instrument, China). The heart was then perfused with nominally Tyrode's buffer containing collagenase II (0.17 mg/ml), collagenase IV (0.10 mg/ml), bovine serum albumin (1.0 mg/ml), and 50 μM CaCl_2_ until it became soft and spongy. The myocytes were mechanically dispersed and stored in the Kraft-Brühe (K-B) buffer. The concentration of Ca^2+^ was raised from 0.2 mM to 1.5 mM using a 4-step ladder. The freshly isolated cardiomyocytes were stored at room temperature (25°C) for Ca^2+^ imaging.

### Ca^2+^ Imaging in Freshly Isolated Left Ventricular Myocytes

The intracellular Ca^2+^ levels were monitored using a fluorescent calcium probe Fluo4-AM, as previously described (Chen et al., 2014). Briefly, freshly isolated cardiomyocytes were seeded in a dish coated with laminin and incubated for 30 min at 37°C to adhere to the bottom of the dish. The supernatant was removed, and cells were washed three times with a modified K-H buffer, and then the cells were incubated with 5 μM Fluo4-AM in the dark for 30 min; the Fluo4-AM-containing buffer was then removed, and cells were washed three times with a modified K-H buffer. The LV myocytes were imaged with the Nikon A1+ confocal microscope (Nikon, Tokyo, Japan) equipped with a 20X objective. The fluorescence intensity was detected with a 525 ± 25 nm filter, and the excitation wavelength was 488 nm. All Ca^2+^-imaging experiments were carried out at room temperature.

### Chemicals and Solutions

Berberine chloride and sodium pentobarbital were purchased from Sigma-Aldrich (St. Louis, MO, the United States), and epinephrine bitartrate was from Adamas (Shanghai, China), and they were dissolved in deionized water. Nifedipine (Sigma-Aldrich, St. Louis, MO, the United States), FPL-64716 (Aladdin, Shanghai, China), and Fluo4-AM (Dojindo, Kyushu, Japan) were dissolved in DMSO, and the final concentration of DMSO in the buffer did not exceed 0.1%. All other chemicals were purchased from Sigma-Aldrich (St. Louis, MO, the United States).

The K-H buffer for Langendorff-perfused rat heart recording was prepared freshly and contained the following (in mM): NaCl 119.0, NaHCO_3_ 25.0, KCl 4.6, D-(+)-glucose 11.0, CaCl_2_·2H_2_O 1.5, MgSO_4_·7H_2_O 1.6, and KH_2_PO_4_ 1.2.

The K-H buffer for isometric tension recording experiment contained the following (in mM): NaCl 119.0, NaHCO_3_ 25.0, KCl 4.6, D-(+)-glucose 11.0, CaCl_2_·2H_2_O 1.5, MgSO_4_·7H_2_O 1.6, KH_2_PO_4_ 1.2, and sodium pyruvate 1.0.

The Ca^2+^-free K-H buffer was K-H buffer, excluding CaCl_2_·2H_2_O and containing 1 mM ethylene glycol-bis (2-aminoethylether)-N, N, N', N'-tetraacetic acid.

The Cs^+^-containing Na^+^-free K-H buffer was prepared freshly and contained the following (in mM): CsCl 119.0, CsHCO_3_ 25.0, KCl 5.8, D-(+)-glucose 11.0, CaCl_2_·2H_2_O 1.5, and MgCl_2_ 1.6.

The N-methyl-D-glucamine (NMDG)-containing Na^+^-free K-H buffer contained (in mM): NMDG 119.0, CsHCO_3_ 25.0, KCl 5.8, D-(+)-glucose 11.0, CaCl_2_·2H_2_O 1.5, and MgCl_2_ 1.6.

The pH of all buffers was adjusted to 7.40 with a 95% O_2_/5% CO_2_ mixture except those explicitly indicated.

The nominally Ca^2+^-free Tyrode's buffer for the isolation of LV myocytes contained the following (in mM): NaCl 140.0, KCl 5.4, MgCl_2_ 1.0, D-(+)-glucose 10.0, and 4-(2-hydroxyethyl)-1-piperazinethanesulfonic acid (HEPES) 10.0; pH was adjusted to 7.35 with NaOH.

The K-B buffer for maintaining isolated LV myocytes contained the following (in mM): KOH 80.0, KCl 40.0, L-glutamic acid 50.0, KH_2_PO_4_ 25.0, taurine 20.0, HEPES 10.0, ethylene glycol-bis (2-aminoethylether)-N, N, N', N'-tetraacetic acid 0.1, and MgSO_4_ 3.0, D-(+)-glucose 10.0; pH was adjusted to 7.40 with KOH.

The modified K-H buffer for Ca^2+^ imaging experiments contained the following (in mM): NaCl 125.0, KCl 4.0, CaCl_2_ 1.5, MgCl_2_ 1.0, D-(+)-glucose 10.0, HEPES 25.0, and NaH_2_PO_4_ 1.2; the Ca^2+^-free modified K-H buffer was the modified K-H buffer excluding CaCl_2_·2H_2_O and containing 1 mM ethylene glycol-bis (2-aminoethylether)-N, N, N', N'-tetraacetic acid; the low Na^+^ modified K-H buffer contained the following (in mM): NaCl 62.5, NMDG 62.5, KCl 4.0, CaCl_2_ 1.5, MgCl_2_ 1.0, D-(+)-glucose 1.0, HEPES 25.0, and NaH_2_PO_4_ 1.2; pH was adjusted to 7.30 with 12 mM HCl.

All buffers were filtered through a membrane with a pore size of 0.22 µm.

### Data Analysis and Statistics

LabChart 8.1.5 (ADInstruments, Australia) was used to analyze the parameters of perfused heart function and developed tension. NIS-Elements 4.3 (Nikon, Japan) and Clampfit 10.5 (Molecular Devices, the United States) was used to analyze the intracellular Ca^2+^ level. The 5-min recordings before each addition of berberine were analyzed. The parameters were normalized to the recording before the first addition of berberine (taken as 100%) in Langendorff-perfused heart recordings. The isolated LVM strip tension in the 5-min recording before the addition of any compound was taken as the basal level (0%), and the effect of vehicle, berberine, or inhibitors was normalized to the basal level. The average fluorescence intensity in the last 3 min of the 15-min perfusion of vehicle, berberine, or inhibitors was analyzed and normalized to that of the last 3-min recording with the modified K-H buffer (taken as 100%). Statistical analysis was performed with GraphPad Prism 7.02 (GraphPad Software, La Jolla, CA). All values are summarized as means ± SEM; n = the number of cells, LVM strips, and N = the number of rats. Statistical significance was performed with the two-way analysis of variance (ANOVA) with Sidak's multiple comparisons test or Tukey's multiple comparisons test, or the unpaired Student's t-test.

## Results

### Berberine Increased the Cardiac Contractility in Langendorff-Perfused Isolated Rat Hearts

**Figure 1:**
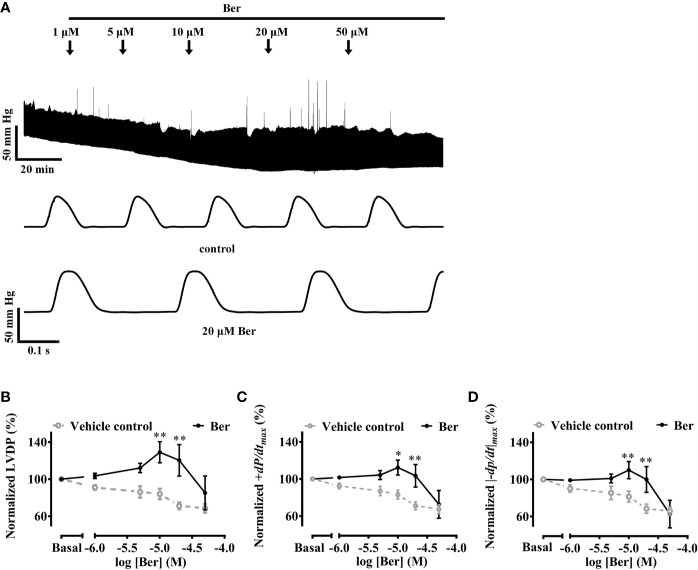
Berberine increased the capability of Langendorff-perfused isolated rat hearts for contraction. **(A)** An original recording showing that the effect of berberine on left ventricular pressure (LVP) of an isolated rat heart. **(B–D)** Summary data showing the effects of berberine on LVDP, +*dp/dt_max_* and |−*dp/dt*|*_max_*, respectively. The numbers of repeats are as follows: vehicle control group, N = 6; Ber-treated group, N = 6; N is the number of rats. Ber stands for berberine. *p < 0.05, **p < 0.01 vs. vehicle control group; two-way ANOVA with Sidak's multiple comparisons test.

The Langendorff-perfused isolated-heart recordings provide information on heart functions without interference from the sympathetic nervous system (Bell et al., 2011). The isolated hearts were perfused for at least 30 min until they reached a stable state before the addition of berberine in the buffer (**Figure 1A**). The left ventricular pressure (LVP) wasW continuously recorded for at least 30 min at each concentration of berberine until it reached a stable level, which was defined as the variation of LVP amplitude was less than 10% within 5 min.

The LVDP, +*dp/dt_max_* and |−*dp/dt*|*_max_* are important parameters used for the evaluation of heart contractility (Jiang et al., 2014; Tilley et al., 2014; Huang et al., 2019). These three parameters increased following the perfusion of berberine (10–20 µM) while they decreased with time in the absence of berberine (**Figures 1B–D**). Following the perfusion of berberine (10 µM), LVDP, +*dp/dt_max_*, and |−*dp/dt*|*_max_* increased to 129.0 ± 11.4%, 112.3 ± 8.0%, and 110.0 ± 9.3% of the basal levels, respectively; they decreased with time to 84.0 ± 5.8%, 83.1 ± 4.8%, and 81.5 ± 5.8% of the basal levels in the absence of berberine, respectively. The results indicated that berberine could improve the heart function by enhancing contractility of the left ventricle. The LV contractility is crucial for heart function. Next, we investigated the effect of berberine on the contractile properties of LVM strips by using isometric tension recordings.

### Berberine Increased the Isometric Tension of LVM Strips of Rat Hearts

**Figure 2:**
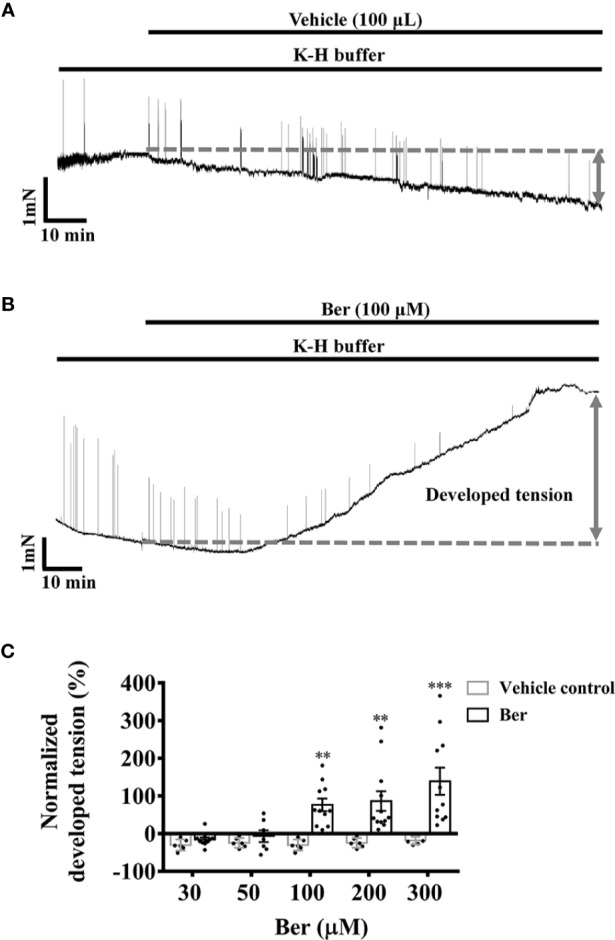
Berberine increased the tension of freshly isolated left ventricular muscle (LVM) strips**. (A, B)** Original isometric tension recordings showing that berberine (100 µM) increased the tension of LVM strips. **(C)** Summary data showing that berberine increased the tension of LVM strips in a concentration-dependent manner. The numbers of repeats are as follows: 30 µM Ber, n = 10, N = 10; 50 µM Ber, n = 7, N = 7; 100 µM and 300 µM Ber, n = 11, N = 11; 200 µM Ber, n = 12, N = 12; n and N are the numbers of LVM strips and rats, respectively. Ber stands for berberine. **p < 0.01, ***p < 0.001 vs. vehicle control group; two-way ANOVA with Sidak's multiple comparisons test.

In the isometric tension recordings, the initial tension of the strips was set to 5 mN, which declined with time; berberine reversed the decline and further increased the tension of LVM strips in a concentration-dependent manner. The isometric tension increased by 76.4 ± 16.7% with the treatment of 100 µM berberine, while it decayed with time by 18.0 ± 4.3% in the absence of berberine (**Figure 2**). These suggested that berberine can increase the contractile force of LV myocardium.

**Figure 3:**
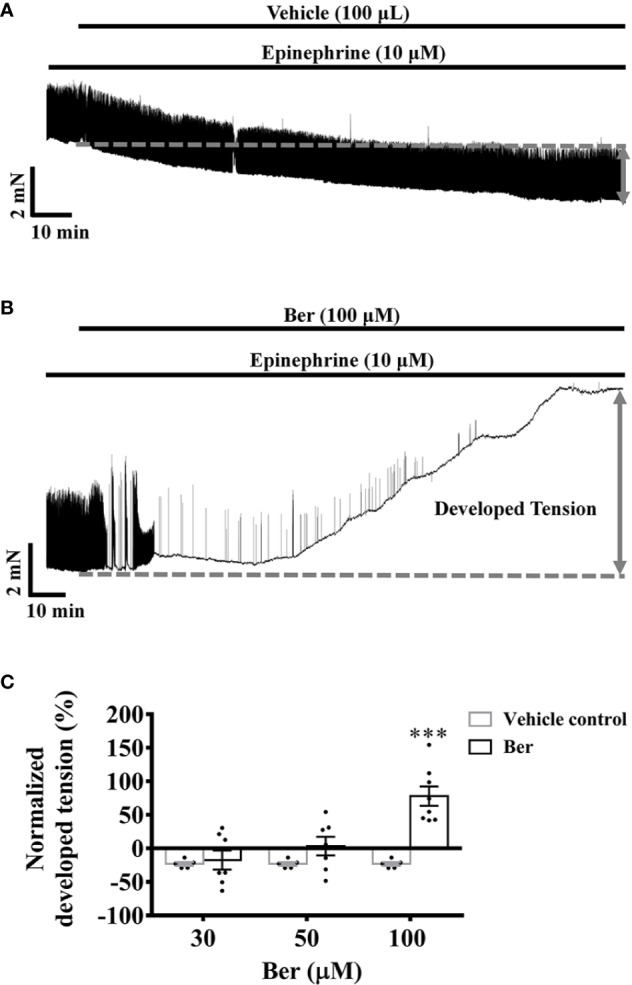
Berberine increased the tension of the freshly isolated left ventricular muscle (LVM) strips pretreated with epinephrine. **(A, B)** Original isometric tension recordings showing that berberine (100 µM) increased the tension and abolished the epinephrine-evoked phasic contractions in LVM strips. **(C)** Summary data showing that the effect of berberine on the tension of the LVM strips pretreated with epinephrine. The numbers of repeats are as follows: 30 µM and 50 µM Ber, n = 7, N = 7; 100 µM Ber, n = 8, N = 8; n and N are the numbers of LVM strips and rats, respectively. Ber stands for berberine. ***p < 0.001 vs. vehicle control group; two-way ANOVA with Sidak's multiple comparisons test.

Epinephrine plays an essential role in the fight-or-flight response by increasing the output of the heart *via* the activation of β-adrenergic receptors. Under pathophysiological conditions, in the case of heart failure, the excessive activation of β-adrenergic receptors causes sustained elevation of cAMP, induces tachycardia and arrhythmic contractions, and increases cardiac workload (Galindo-Tovar and Kaumann, 2008; Johnson and Antoons, 2018). Epinephrine (10 µM) induced phasic contractions in the isolated LVM strips. In the presence of epinephrine, the tension of LVM strips decreased with time, while the amplitude of the evoked phasic contractions did not change (**Figure 3A**). Following epinephrine, the subsequent addition of berberine increased the tension and eliminated the evoked phasic contractions in a concentration-dependent manner (**Figures 3B, C**). Berberine (100 μM) increased the tension by 77.9 ± 14.4%, while it decreased by 23.1 ± 2.8% with time in the absence of berberine (**Figure 3**). These indicated that berberine could enhance the contractile force and eliminate the epinephrine-evoked phasic contractions in the LVM.

**Figure 4:**
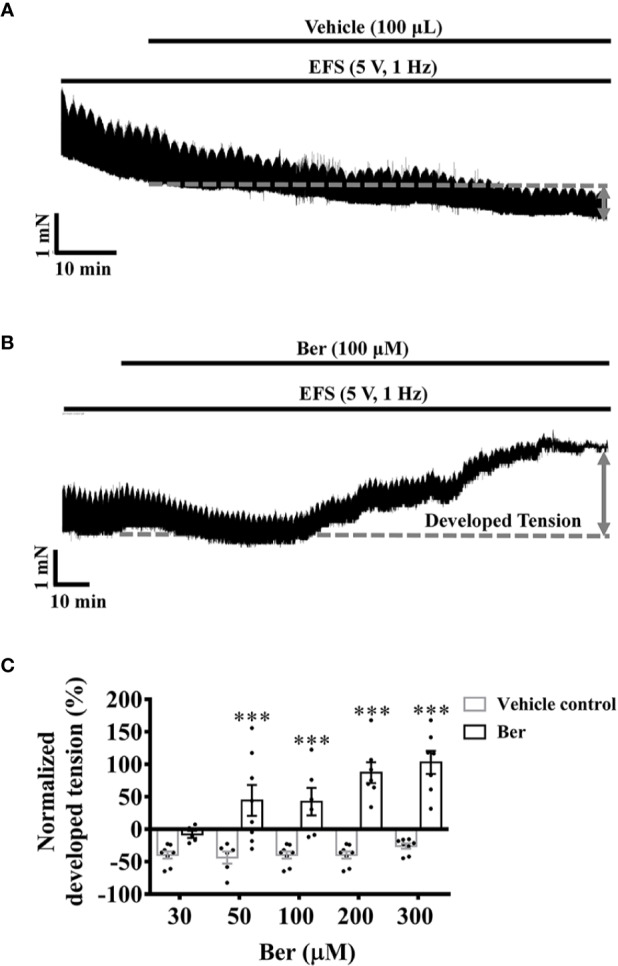
Berberine increased the tension of the electrical field stimulation (EFS**)-**paced left ventricular muscle (LVM) strips**. (A, B)** Original isometric tension recordings showing that berberine (100 µM) increased the tension of the EFS-paced LVM strips (5 V, 1 Hz). **(C)** Summary data indicating that berberine increases the tension of the EFS-paced LVM strips in a concentration-dependent manner. The numbers of repeats are as follows: 30 µM Ber, n = 5, N = 5; 50 µM Ber, n = 8, N = 8; 100 µM Ber, n = 6, N = 6; 200 µM Ber and 300 µM Ber, n = 7, N = 7; n and N are the numbers of LVM strips and rats, respectively. Ber stands for berberine. ***p < 0.001 vs. vehicle control group; two-way ANOVA with Sidak's multiple comparisons test.

The action potentials generated by the sinoatrial node transmit through the cardiac electrical conduction system to cause myocardial contraction (Nakao et al., 2015). The electrical field stimulations (EFS) induced phasic contractions in LVM strips, but it did not prevent the decline in the tension of LVM strips with time (**Figure 4A**). The addition of berberine prevented the tension decrease and further increased the tension of EFS-paced LVM strips (**Figure 4B**). The tension increased by 42.5 ± 21.0% following the addition of 100 μM berberine, but it decreased by 39.7 ± 5.5% with time in the absence of berberine (**Figure 4**). These data suggested that berberine could improve heart function by increasing the isometric tension of the ventricular muscle. The cardiac contractility relies on the intracellular Ca^2+^ level of the cardiomyocytes, so we next determined the effect of berberine on the intracellular Ca^2+^ level of LV myocytes.

### Berberine Increased the Intracellular Ca^2+^ Level of LV Myocytes in a Concentration-Dependent Manner

**Figure 5:**
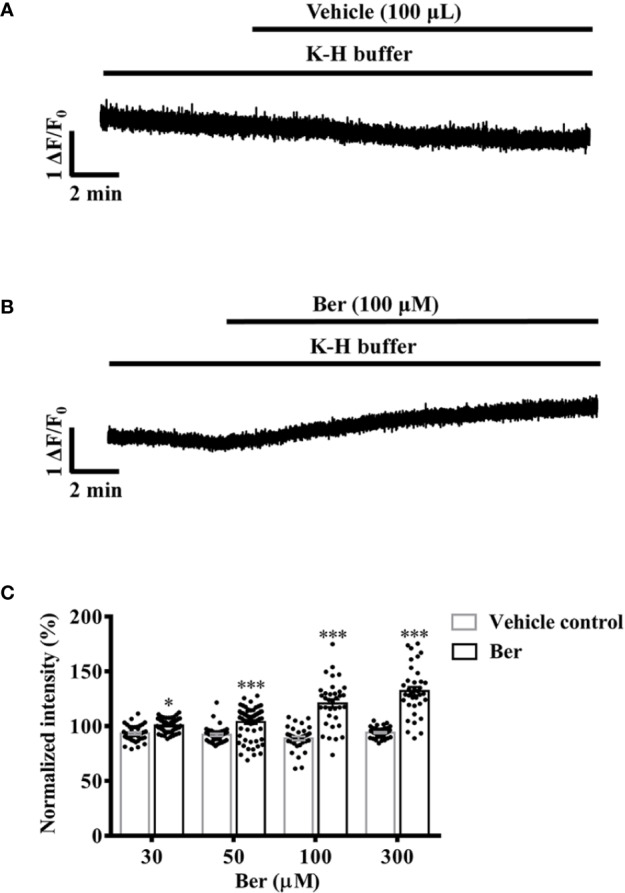
Berberine concentration-dependently increased the intracellular Ca^2+^ level of freshly isolated left ventricular (LV) myocytes. **(A, B)** Original Ca^2+^ imaging recordings showing that berberine (100 µM) increased the intracellular Ca^2+^ level of freshly isolated LV myocytes. **(C)** Summary data showing that berberine concentration-dependently increased the intracellular Ca^2+^ level. The numbers of repeats are as follows: 30 µM Ber, n = 54, N = 3; 50 µM Ber, n = 65, N = 3; 100 µM Ber, n = 35, N = 3; 300 µM Ber, n = 36, N = 3; n and N are the numbers of LV myocytes and rats, respectively. Ber stands for berberine. *p < 0.05, ***p < 0.001 vs. vehicle control group; two-way ANOVA with Sidak's multiple comparisons test.

The increase of the intracellular Ca^2+^ level is the key to cardiac contractions (Bers, 2002; Eisner et al., 2017). In the range of 30–300 μM, berberine increased the intracellular Ca^2+^ level of freshly isolated LV myocytes (**Figure 5C**). The fluorescence intensity of Fluo4-Ca^2+^ increased to 120.8 ± 21.1% with the treatment of 100 µM berberine, while it decayed with time to 88.83 ± 10.71% in the absence of berberine (**Figure 5**). These results indicated the berberine might elicit the positive inotropic effect by elevating the intracellular Ca^2+^ level of LV myocytes.

### The Berberine-Induced Tension Increase in LVM Strips Was Extracellular Ca^2+^-dependent

**Figure 6:**
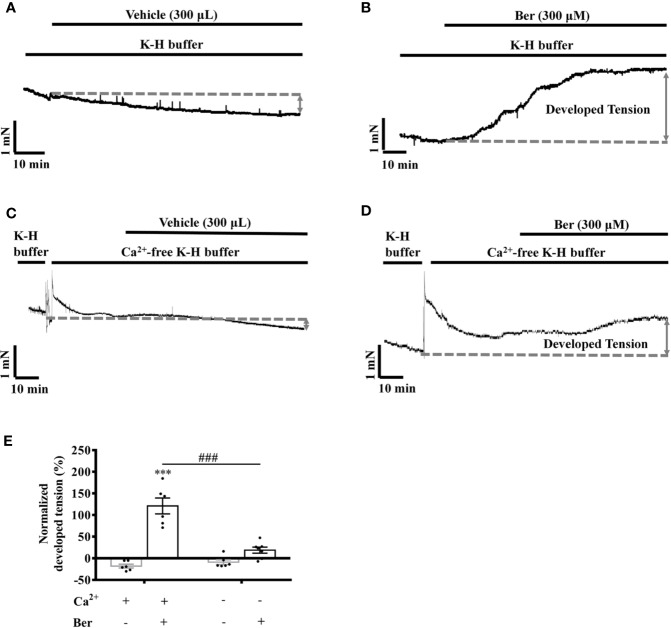
The removal of extracellular Ca^2+^ hindered the berberine-induced tension increase in left ventricular muscle (LVM) strips. **(A–D)** Original isometric tension recordings of the LVM strips treated with vehicle **(A)**, 300 μM Ber **(B)**; vehicle **(C)** and 300 μM Ber **(D)** under the Ca^2+^-free condition, respectively. **(E)** Summary data showing that the removal of extracellular Ca^2+^ significantly reduced the ability of berberine to increase the tension of LVM strips. The numbers of repeats are as follows: vehicle control of 300 µM Ber, n = 6, N = 6; 300 µM Ber, n = 6, N = 6; vehicle control of 300 µM Ber in the Ca^2+^-free Krebs-Hensseleit (K-H) buffer, n = 6, N = 6; 300 µM Ber in the Ca^2+^-free K-H buffer, n = 7, N = 7; n and N are the numbers of LVM strips and rats, respectively. Ber stands for berberine. ***p < 0.001 vs. vehicle control group; ^###^*p* < 0.001 vs. Ber-treated group in the normal K-H buffer; two-way ANOVA with Tukey's multiple comparisons test.

Extracellular Ca^2+^ influx contributes to the increase in the intracellular Ca^2+^ level of LV myocytes, which induces cardiac contraction (Bers, 2002; Eisner et al., 2017; Gilbert et al., 2020). To investigate the role of Ca^2+^ influx in the berberine-induced tension increase in LVM strips, we carried out experiments under the Ca^2+^-free condition (Song et al., 2014). The removal of extracellular Ca^2+^ significantly attenuated the berberine-induced tension increase in LVM strips (**Figure 6E**). In the absence of extracellular Ca^2+^, the tension increased by 17.7 ± 19.8% following the addition of berberine (300 µM) (**Figures 6 C, D**), while it increased by 121.0 ± 18.3% in the presence of extracellular Ca^2+^ (1.5 mM) (**Figures 6 A, B**). It suggested that the berberine-induced tension increase in LVM strips is extracellular Ca^2+^-dependent.

### Nifedipine Attenuated the Berberine-Induced Tension Increase in LVM Strips

**Figure 7:**
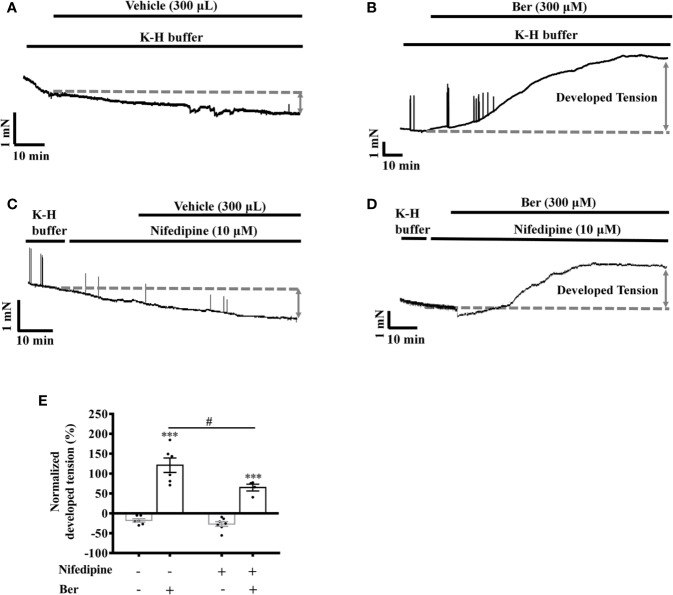
Nifedipine attenuated the berberine-induced tension increase in left ventricular muscle (LVM) strips. **(A–D)** Original isometric tension recordings of the LVM strips treated with vehicle **(A)**, 300 μM Ber **(B)**, 10 μM nifedipine **(C)**, and 300 μM Ber in the presence of 10 μM nifedipine **(D)**, respectively. **(E)** Summary data showing nifedipine hindered the 300 μM berberine-induced tension increase in LVM strips. The numbers of repeats are as follows: vehicle control of 300 µM Ber, n = 6, N = 6; 300 µM Ber, n = 6, N = 6; 10 μM nifedipine, n = 7, N = 7; 300 µM Ber plus 10 μM nifedipine, n = 5, N = 5; n and N are the numbers of LVM strips and rats, respectively. Ber stands for berberine. ***p < 0.001 vs. vehicle control group; ^#^*p* < 0.05, vs. 300 μM Ber-treated group; two-way ANOVA with Tukey's multiple comparisons test.

LTCCs are the major Ca^2+^ channels that allow extracellular Ca^2+^ influx in LV myocytes (Jaleel et al., 2008). The selective LTCC inhibitor nifedipine (10 µM) can greatly block the channels (Carre et al., 2014). Berberine (300 µM) increased the tension in LVM strips by 121.0 ± 18.3% and 63.6 ± 7.5% in the absence and presence of nifedipine (10 µM), respectively (*p* < 0.05, **Figures 7B, D, E**).

### Berberine Induced an Additional Increase in the Tension of LVM Strips in the Presence of LTCC Opener FPL-64716

**Figure 8:**
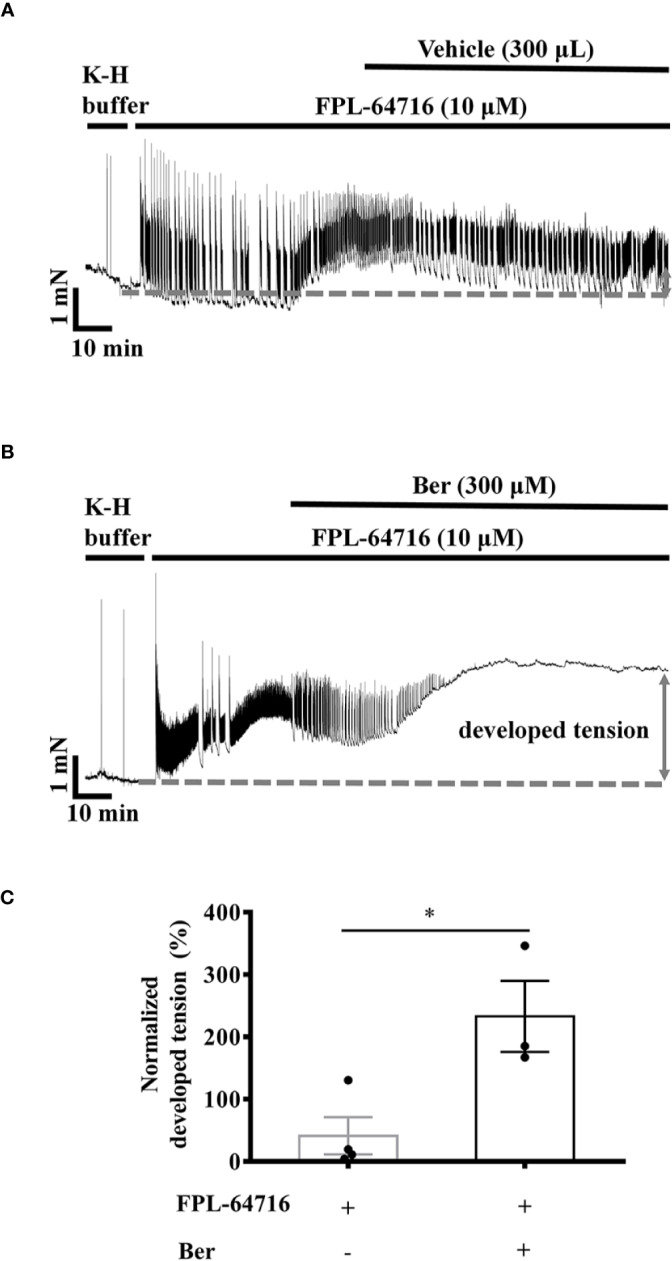
Berberine increased the tension of the freshly isolated left ventricular muscle (LVM) strips pretreated with FPL-64716. (A, B) Original isometric tension recordings of the LVM strips treated with 10 µM FPL-64716 **(A)** and 300 μM berberine in the presence of 10 µM FPL-64716 **(B)**. **(C)** Summary data showing berberine induced an additional increase in the tension of the LVM strips pretreated with FPL-64716. The numbers of repeats are as follows: 10 µM FPL-64716, n = 4, N = 3; 300 µM Ber plus 10 μM FPL-64716, n = 3, N = 3; n and N are the numbers of LVM strips and rats, respectively. Ber stands for berberine. *p < 0.05 vs. 10 µM FPL-64716-treated group; two-tailed unpaired Student's t-test.

The LTCC opener FPL-64716 enhances Ca^2+^ influx (Kang et al., 2012). FPL-64716 (10 μM) increased the tension of LVM strips by 105.1 ± 70.8%, and the subsequent addition of berberine induced a further increase by a total of 232.9 ± 56.9% (**Figure 8B**). It increased by 41.3 ± 29.9% in the vehicle control group (**Figure 8A**). These results indicated that the positive inotropic effects of berberine were LTCC-independent. To directly investigate the effect of berberine on the Ca^2+^ influx, we next carried out experiments with isolated LV myocytes under the Ca^2+^-free condition or in the presence of the LTCC blocker nifedipine.

### Berberine Increased the Intracellular Ca^2+^ Level of LV Myocytes Dependent on the Extracellular Ca^2+^

**Figure 9:**
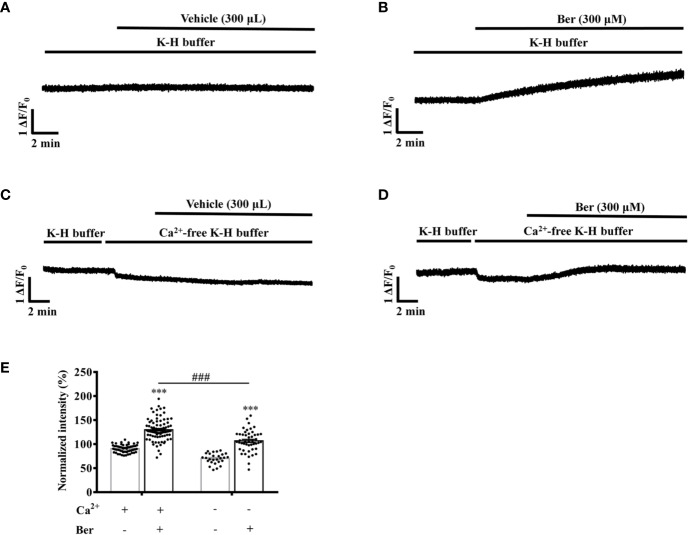
The removal of extracellular Ca^2+^ hindered the berberine-induced increase in the intracellular Ca^2+^ level of freshly isolated LV myocytes. **(A–D)** Original Ca^2+^ imaging recordings of the LV myocytes treated with vehicle **(A)** and 300 μM berberine **(B)**; vehicle **(C)** and 300 μM berberine **(D)** in the Ca^2+^-free modified Krebs-Hensseleit (K-H) buffer, respectively. **(E)** Summary data showing that the removal of extracellular Ca^2+^ significantly hindered the 300 μM berberine-induced increase in the intracellular Ca^2+^ level of LV myocytes. The numbers of repeats are as follows: vehicle control of 300 µM Ber, n = 57, N = 3; 300 µM Ber, n = 83, N = 3; vehicle control of 300 µM Ber in the Ca^2+^-free modified K-H buffer, n = 27, N = 3; 300 µM Ber in the Ca^2+^-free modified K-H buffer, n = 47, N = 3; n and N are the numbers of LV myocytes and rats, respectively. Ber stands for berberine. ***p < 0.001 vs. vehicle control group; ^###^*p* < 0.001 vs. Ber-treated group in the normal modified K-H buffer; two-way ANOVA with Tukey's multiple comparisons test.

Berberine (300 μM) increased the fluorescence intensity of Fluo4-Ca^2+^ of the isolated LV myocytes to 130.0 ± 2.5% in the extracellular buffer containing 1.5 mM Ca^2+^ (**Figures 9A, B**). The removal of extracellular Ca^2+^ significantly attenuated the berberine-induced fluorescence increase to 106.7 ± 3.3% (**Figures 9C–E**). These results indicated that the berberine-induced intracellular Ca^2+^ elevation was extracellular Ca^2+^-dependent and that berberine might enhance the Ca^2+^ influx.

**Figure 10:**
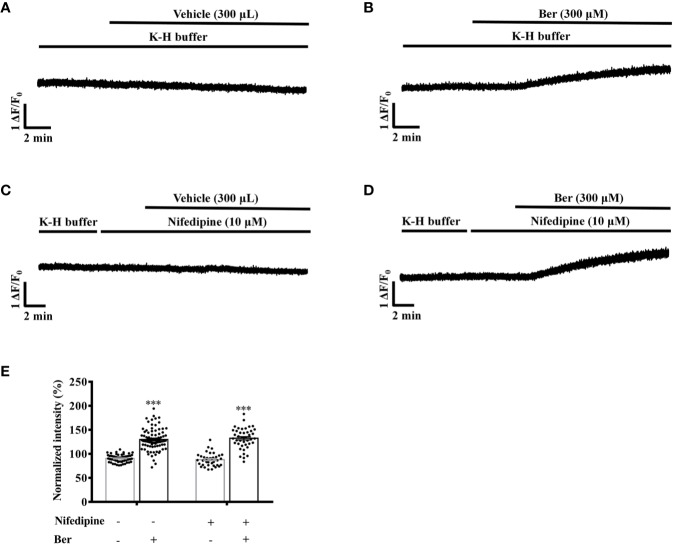
Nifedipine did not influence the 300 μM berberine-induced increase in the intracellular Ca^2+^ level of freshly isolated left ventricular (LV) myocytes. **(A–D)** Original Ca^2+^ imaging recordings of the LV myocytes treated with vehicle **(A)**, 300 μM Ber **(B)**, 10 μM nifedipine **(C)** and 300 μM Ber in the presence of 10 μM nifedipine **(D)**. **(E)** Summary data showing nifedipine did not affect the 300 μM berberine-induced increase in the intracellular Ca^2+^ level of LV myocytes. The numbers of repeats are as follows: vehicle control of 300 µM Ber, n = 57, N = 4; 300 µM Ber, n = 83, N = 4; 10 μM nifedipine, n = 37, N = 4; 300 µM Ber plus 10 μM nifedipine, n = 46, N = 4; n and N are the numbers of LV myocytes and rats, respectively. Ber stands for berberine. ***p < 0.001 vs. vehicle control group; two-way ANOVA with Tukey's multiple comparisons test.

Next, we investigated the role of LTCCs in the berberine-induced increase in the intracellular Ca^2+^ level. In the presence of nifedipine, berberine increased the fluorescence intensity of Fluo4-Ca^2+^ to 130.7 ± 3.2% of the basal level in LV myocytes. It rose to 130.0 ± 2.5% in the absence of nifedipine (**Figures 10B, D**). These suggested that berberine increases the intracellular Ca^2+^ concentration also by enhancing the Ca^2+^ entry *via* an LTCC-independent pathway. The Na^+^ is also critical for regulating the excitation-contraction coupling. Next, we determined the role of the extracellular Na^+^ in the berberine-induced tension increase in the LVM strips.

### Berberine Increased the Tension in LVM Strips Dependent on the Extracellular Na^+^

**Figure 11:**
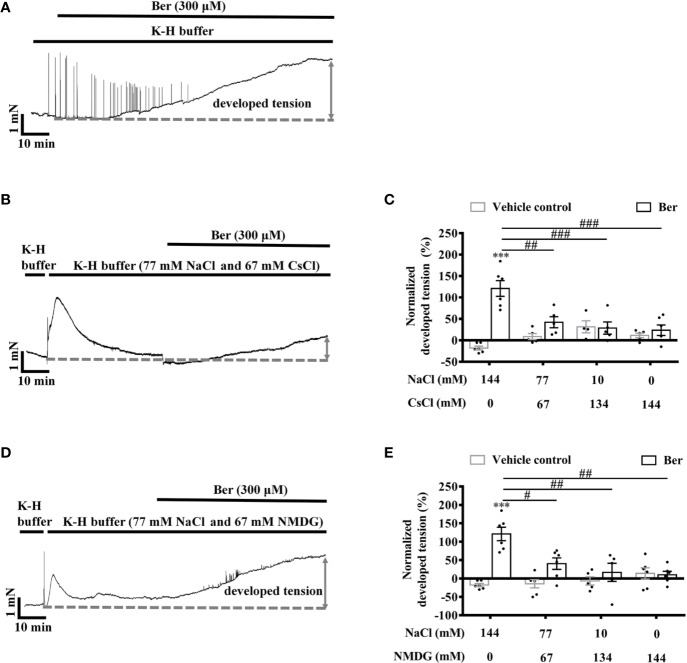
The berberine-induced tension increase in left ventricular muscle (LVM) strips was extracellular Na^+^-dependent**. (A, B, D)** Original isometric tension recordings of the LVM strips treated with 300 μM berberine **(A)**, and 300 μM berberine under the low Na^+^ condition where NaCl of the Krebs-Hensseleit (K-H) buffer was replaced with CsCl **(B)** or with NMDG **(D)**. **(C, E)** Summary data indicating that the 300 µM berberine-induced tension increase has a positive correlation with the concentrations of extracellular NaCl. The numbers of repeats are as follows: experiments with the normal K-H buffer, 300 µM Ber, n = 6, N = 6. Experiments with the K-H buffer in which NaCl was replaced with CsCl: 300 µM Ber in the buffer containing 77 or 10 mM NaCl, n = 5, N = 5; 300 µM Ber in the buffer containing 0 mM NaCl, n = 6, N = 6. Experiments with the K-H buffer in which NaCl was replaced with NMDG: 300 µM Ber in the buffer containing 77 or 0 mM NaCl, n = 6, N = 6; 300 µM Ber in the buffer containing 10 mM NaCl, n = 5, N = 5. n and N are the numbers of LVM strips and rats, respectively. Ber stands for berberine. ***p < 0.001 vs. vehicle control group; ^#^*p <* 0.05, ^##^*p <* 0.01, ^###^*p <* 0.001 vs. Ber-treated group in the normal K-H buffer; two-way ANOVA with Tukey's multiple comparisons test.

To determine the role of extracellular Na^+^ in the berberine-induced tension increase in LVM strips, Cs^+^, and NMDG were used to replace extracellular Na^+^, respectively (Xin et al., 2005). The lowering of extracellular Na^+^ caused a transient increase in the tension of LVM strips (**Figures 11B, D**). The subsequent addition of berberine induced a smaller increase in the tension of LVM strips in the Na^+^-free K-H buffer than that in the standard K-H buffer containing 144 mM NaCl. The berberine-induced tension increased with the concentration of Na^+^ rising in the buffer (**Figures 11C, E**). These data indicated that the extracellular Na^+^ is essential for the tension increase in LVM strips caused by berberine.

### The Berberine-Induced Increase in the Intracellular Ca^2+^ Level of LV Myocytes Was Extracellular Na^+^-Dependent

**Figure 12:**
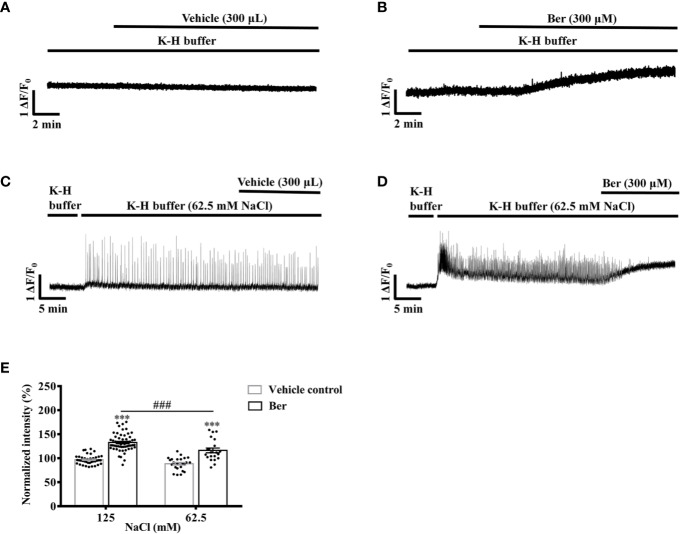
The berberine-induced increase in the intracellular Ca^2+^ level of left ventricular (LV) myocytes was extracellular Na^+^-dependent. **(A–D)** Original Ca^2+^ imaging recordings of the LV myocytes treated with vehicle **(A)** and 300 μM Ber **(B)**; vehicle **(C)** and 300 μM Ber **(D)** in the modified Krebs-Hensseleit (K-H) buffer containing 62.5 mM NaCl and 62.5 mM NMDG, respectively. (B) Summary data indicating that the berberine-induced increase in the intracellular Ca^2+^ level in a buffer containing 62.5 mM NaCl was smaller than that in a buffer containing 125 mM NaCl. The numbers of repeats are as follows: vehicle control of 300 µM Ber, n = 42, N = 3; 300 µM Ber, n = 58, N = 3; vehicle control of 300 µM Ber in the K-H buffer containing 62.5 mM NaCl, n = 24, N = 3; 300 µM Ber in the K-H buffer containing 62.5 mM NaCl, n = 21, N = 3; n and N are the numbers of LV myocytes and rats, respectively. Ber stands for berberine. ***p < 0.001 vs. vehicle control group; *^###^p <* 0.001 vs. Ber-treated group in the modified K-H buffer containing 125 mM NaCl; two-way ANOVA with Tukey's multiple comparisons test.

To further investigate the role of extracellular Na^+^ in the berberine-induced tension increase in LVM strips, we examined the impact of extracellular Na^+^ on the berberine-induced rise in the intracellular Ca^2+^ level of LV myocytes. Berberine (300 μM) raised the fluorescence intensity of Fluo4-Ca^2+^ to 132.5 ± 18.1% of the basal level in myocytes perfused with the modified K-H buffer containing 125 mM NaCl (**Figure 12B**). We perfused the myocytes with a modified K-H buffer containing 62.5 mM NaCl and 62.5 mM NMDG. It caused a transient increase in the fluorescence intensity of Fluo4-Ca^2+^. Following a 30-min equilibration, the subsequent addition of berberine (300 μM) elevated the fluorescence intensity of Fluo4-Ca^2+^ to 116.2 ± 22.5% of the basal level (**Figure 12D**). The berberine-induced increase in the fluorescence intensity in LV myocytes was significantly less in the 62.5 mM-NaCl K-H buffer than that in the standard, modified K-H buffer containing 125 mM NaCl (**Figure 12E**). These data indicated that the elevation in the intracellular Ca^2+^ level by berberine was extracellular Na^+^-dependent.

## Discussion

Here we reported that berberine significantly increased the LVDP, +*dp/dt*_max_, and the |−*dp/dt*|*_max_* in Langendorff-perfused isolated rat hearts. Berberine increased the tension of the isolated LVM strips and the intracellular Ca^2+^ level of isolated LV myocytes. The removal of extracellular Ca^2+^ or lowering extracellular Na^+^ reduced the berberine-induced increases in both LVM tension and the Ca^2+^ levels in LV myocytes. These results suggested that berberine increased the intracellular Ca^2+^ concentration of LV myocytes dependent on the extracellular Ca^2+^ and Na^+^. It consequently exerted its positive inotropic effect on the rat heart.

A clinical study showed that oral administration of berberine (1.2–2.0 g/d) increased LV ejection fraction in patients with chronic congestive heart failure who also took medications of angiotensin-converting enzyme inhibitors, digoxin, diuretics, and nitrates (Zeng et al., 2003). The LV ejection fraction increase in patients with plasma berberine concentrations higher than 0.1 mg/L (~0.3 μM) was more significant than in those with concentrations lower than 0.1 mg/L (Zeng et al., 2003). In the concentration range from 10 to 20 μM, berberine exhibited positive inotropic effects on the isolated rat hearts (**Figures 1B–D**). The sufficient concentrations in the *in vitro* study were higher than those observed in the clinical studies (Zeng and Zeng, 1999; Zeng et al., 2003).

Besides, Tan investigated the tissue distribution of berberine and its metabolites in rats with a single oral dose of 200 mg/kg (Tan et al., 2013). The time for berberine to reach its maximal concentrations in the plasma (~76.8 nM) and heart (~2 ng/g) were 1.33 ± 0.29 and 24 h, respectively. Berberrubine, one of the primary active metabolites of berberine, was also found in the heart (Li et al., 2011; Tan et al., 2013). The metabolite of berberine might contribute to the therapeutic effects of berberine as well. The significant decrease in resting systolic and diastolic blood pressures might also be beneficial to the heart failure patients treated with berberine (Zeng et al., 2003).

Berberine increased both the tension of LVM strips and the intracellular Ca^2+^ level of LV myocytes in the concentration range from 30 or 50 to 300 μM (**Figures 2** and **5**). The effective concentrations of berberine in these two preparations were higher than those (10–20 μM) in the Langendorff-perfused whole hearts. A previous study also showed that berberine (10–300 μM) increased the developed force in the left atrium of guinea pigs (Shaffer, 1985). These suggested that the impact of berberine on the cardiac function in patients was a systemic phenomenon rather than only *via* targeting the myocytes. Berberine increased the intracellular Ca^2+^ level of LV myocytes in a concentration-dependent manner, which reached the maximal level at 300 µM (**Figure 5**). This response was consistent with the maximal tension increase induced by 300 µM berberine in LVM strips (**Figures 2** and **5**). Therefore, we selected 300 µM berberine in the final study to determine the underlying mechanism.

The intracellular Ca^2+^ concentration in LV myocytes is the key for controlling the cardiac contractions, and the Ca^2+^ influx is critical for raising the intracellular Ca^2+^ level (Bers, 2002; Eisner et al., 2017; Gilbert et al., 2020). Berberine elevated the tension of LVM strips and the intracellular Ca^2+^ concentration in the isolated LV myocytes bathed in a buffer containing 1.5 mM Ca^2+^ (**Figures 6B** and **9B**). The removal of Ca^2+^ from the buffer attenuated the functions of berberine (**Figures 6D** and **9D**). These suggested that the berberine-induced tension increase in LVM strips is dependent on the extracellular Ca^2+^, and berberine elevates the intracellular Ca^2+^ level in LV myocytes by enhancing the Ca^2+^ influx.

L-type Ca^2+^ channel (LTCC) is the primary channel for Ca^2+^ entry in cardiac myocytes. Previous studies showed that berberine decreased or increased LTCC currents in guinea pig ventricular cells (Wang and Zheng, 1997; Xu et al., 1997), but had no effect on the LTCC current in cat ventricular cells (Sanchez-Chapula, 1996). Our results showed that the pretreatment with nifedipine did not affect the berberine-induced increase in the intracellular Ca^2+^ level of rat LV myocytes (**Figure 10**). This result indicated that berberine enhanced the Ca^2+^ entry through pathways not dependent on LTCCs.

The LTCC inhibitor nifedipine significantly attenuated the berberine-induced tension increase in the rat LVM strips (**Figure 7**). In the presence of the LTCC opener FPL-64716, berberine caused an additional increase in LVM strip tension (**Figure 8**). Besides, berberine significantly increased the intracellular Ca^2+^ concentration of LV myocytes bathed in the Ca^2+^-free buffer (**Figure 9D**). It indicated the berberine could enhance the Ca^2+^ release from the intracellular stores, but further research is needed to determine the mechanism. These suggested that berberine might also exert a positive inotropic effect through an LTCC-independent mechanism.

Sodium is also a critical cation regulating the excitation-contraction coupling in cardiomyocytes. The positive inotropic effect of berberine on LV strips bathed in the low Na^+^ buffer decreased (**Figure 11**). This phenomenon demonstrated that the berberine-induced tension increase in LVM strips was dependent on the extracellular Na^+^.

The acutely isolated cardiomyocytes are depolarized, and their intracellular Na^+^ level is elevated (Yao et al., 1998; Despa et al., 2002). The Na^+^ influx during the action potential activated reverse NCX and promoted the Ca^2+^ entry in cardiomyocytes and neurons (Czyz and Kiedrowski, 2002; Larbig et al., 2010; Yan et al., 2015). The removal of extracellular Na^+^ inhibits the increase in the intracellular Ca^2+^ level of human epidermoid carcinoma A-431 cells by NaCN (Kiang and Smallridge, 1994). The current study also showed that the reduction in the Na^+^ concentration in the buffer suppressed the elevation of the intracellular Ca^2+^ level in LV myocytes by berberine (**Figure 12**). A previous study showed that berberine increased myocardial contractility and cardiac output *via* interrelated mechanisms, including the lengthening of ventricular action potential duration, which was partially due to stimulating the reverse mode of NCX (Wang and Zheng, 1997; Lau et al., 2001). The data presented here indicated that berberine increased the tension of LVM strips *via* a Na^+^-dependent Ca^2+^ entry, which might be through activating the reverse mode of NCX.

## Conclusion

The current study revealed that berberine elevated the intracellular Ca^2+^ level in LV myocytes by increasing Ca^2+^ influx. It consequently increased the LV contractility. These were extracellular Na^+^-dependent.

## Data Availability Statement

All datasets generated for this study are included in the article/supplementary material.

## Ethics Statement

The animal study was reviewed and approved by College of Pharmaceutical Sciences, Southwest University, Chongqing, China.

## Author Contributions

WX, YW, and JZ contributed to the study design. JZ, YW, and YJ carried out literature research. JZ, YW, JG and YJ performed experiments. WX, JZ, YW and JG contributed to data analysis. WX, JZ, and YW contributed to manuscript preparation and manuscript revision. The first two authors contributed equally to the manuscript.

## Conflict of Interest

The authors declare that the research was conducted in the absence of any commercial or financial relationships that could be construed as a potential conflict of interest.

## Abbreviations

HEPES, 4-(2-hydroxyethyl)-1-piperazinethanesulfonic acid; K-B, Kraft-Brühe; K-H, Krebs-Henseleit; LTCC, L-type Ca^2+^ channel; LV, left ventricular; LVDP, left ventricular developed pressure; LVEDP, left ventricular end-diastolic pressure; LVM, left ventricular muscle; LVP, left ventricular pressure; LVSP, left ventricular systolic pressure; NMDG, N-Methyl-D-glucamine; NCX, Na^+^/Ca^2+^ exchanger.
